# Supplementary material for: B cells inhibit bone formation in rheumatoid arthritis by suppressing osteoblast differentiation
Source: Nat Commun. 2018 Dec 3;9:5127. doi: 10.1038/s41467-018-07626-8 (PMC6277442; doi:10.1038/s41467-018-07626-8)
Supplement: Supplementary file 1 — Supplementary Information [file 41467_2018_7626_MOESM1_ESM.pdf]

Supplementary Information

“B cells inhibit bone formation in rheumatoid arthritis by suppressing osteoblast differentiation”

Sun et al.

Supplementary Methods

1) CCL3-KO mice were obtained from Jackson Laboratories. The presence of the WT CCL3 allele was detected using the WT forward primer 5′-CTTGGGTGGAGAGGCTATTC -3′ and WT reverse primer 5′-AGGTGAGATGACAGGAGATC -3′. The null CCL3 allele was detected using the Mut forward primer 5′- ATGAAGGTCTCCACCACTGC -3′ and the Mut reverse primer 5′- AGTCAACGATGAATTGGCG -3. TNF-Tg male mice and CCL3-KO female mice were crossed to generate TNF-Tg/CCL3+/− double-mutant mice, and TNF-Tg/CCL3+/− males and CCL3-KO females were crossed to generate TNF-Tg/CCL3-KO mice.

2) TNF-KO mice were obtained from Jackson Laboratories and are maintained in the CMSR. The common forward primer for both WT and Mut is 5′-TAGCCAGGAGGGAGAACAGA -3. The presence of the WT TNF allele was detected using the WT reverse primer 5′- AGTGCCTCTTCTGCCAGTTC -3. The null TNF allele was detected using the Mut reverse primer 5′-CGTTGGCTACCCGTGATATT -3′. TNF-Tg male mice and TNF-KO female mice were crossed to generate TNF-Tg/TNF+/− double-mutant mice, and

TNF-Tg/TNF+/− males and TNF-KO females were crossed to generate TNF-Tg/TNF-KO mice.

3) CCL3-KO mice and TNF-KO mice were crossed to generate CCL3+/−/TNF+/− double-mutant mice, and CCL3+/−/TNF+/− males and females were crossed to generate CCL3-KO/TNF-KO mice. TNF-Tg/CCL3-KO male mice and CCL3-KO/TNF-KO female mice were crossed to generate TNF-Tg/CCL3-KO/TNF+/− triple-mutant mice, and TNF-Tg/CCL3-KO/TNF+/− males and CCL3-KO/TNF-KO females were crossed to generate TNF-Tg/CCL3-KO/TNF-KO mice.

4) For the ectopic bone formation assay, MPCs were isolated from WT or GFP transgenic mice and cultured to a third passage. BM B cells from 4-month-old WT, TNF-Tg, TNF-Tg/CCL3-KO, TNF-Tg/TNF-KO, TNF-Tg/CCL3-KO/TNF-KO or mTmG mice were purified with anti-CD19 beads. Gelfoam (Pfizer) was loaded with $1x10^6$ MPCs and $1x10^7$ B cells, and subcutaneously implanted into the dorsal surfaces of 2-month-old NOD-SCID mice. Briefly, after anesthetizing mice, skin was cleaned with iodine and a ~2 cm incision was made perpendicular to the spine

on both sides of the dorsal surface of the mice. Sponges were inserted into the pocket (up to 3 sponges in 1 pocket) and the incision was closed with sutures. Every NOD-SCID mouse carried 6 sponges, including B cells from WT, TNF-Tg, TNF-Tg/CCL3-KO, TNF-Tg/TNF-KO, or TNF-Tg/CCL3-KO/TNF-KO mice plus WT MPCs, or mTmG BM B cells with MPCs from GFP transgenic mice. The implants were harvested 4 weeks after implantation, processed through paraffin and stained with H&E and Goldner's Trichrome.

Supplementary Figures

Supplementary Figure 1

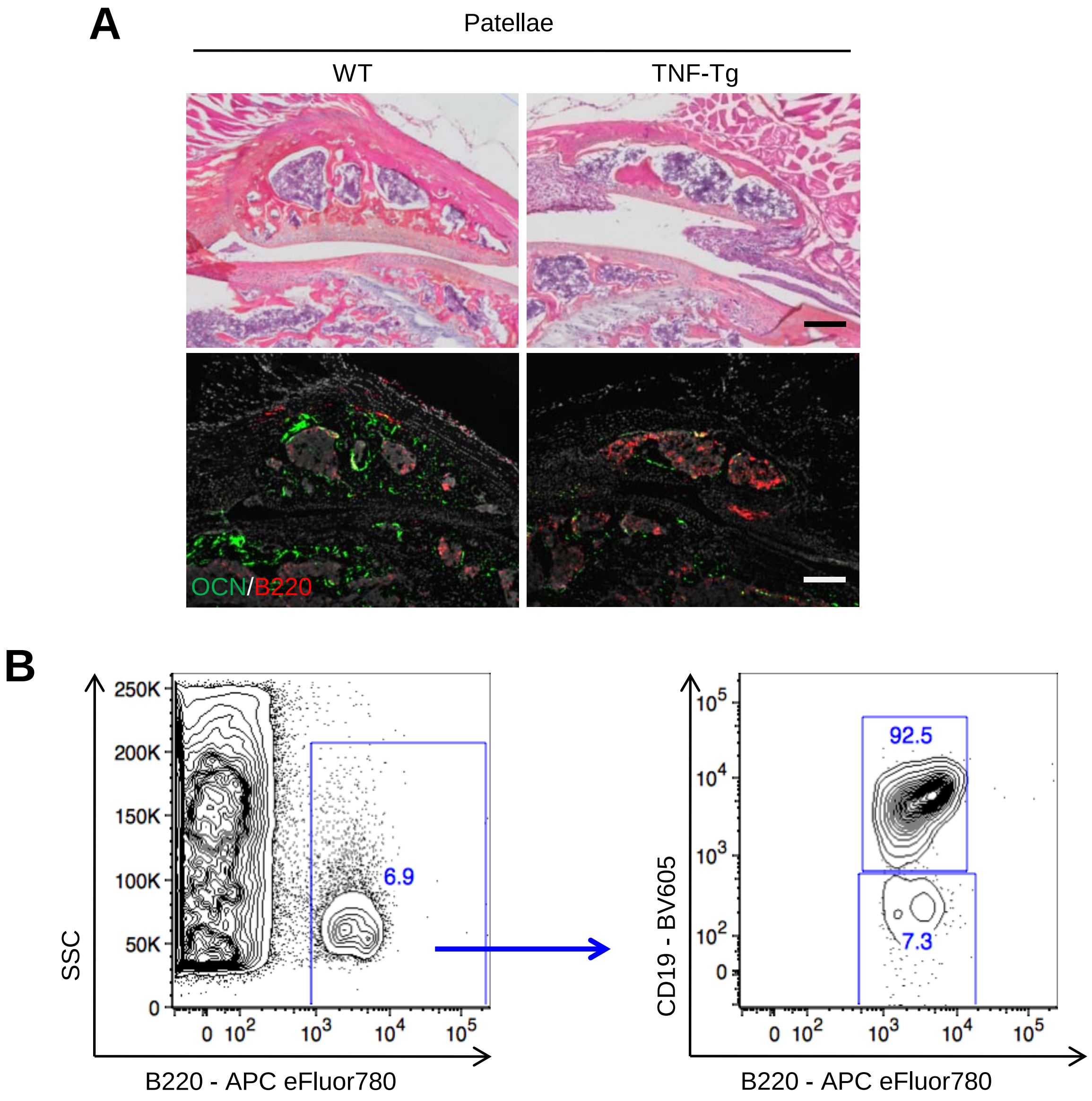

Supplementary Fig. 1. B cell distribution in patellae and subchondral bone marrow/synovial B220+ B cells confirmation.
(**A**) Frozen sections of patellae from a 6-m-old TNF-Tg and WT mice were H&E-stained (upper panel), and adjacent sections (lower panel) were subjected to IF with anti-B220 Ab for B cells (red) and anti-osteocalcin (OCN) Ab for osteoblasts (OBs, green). Bar =200µm. (**B**) Flow cytometry analysis of subchondral bone marrow/synovial (SBM) cells from a TNF-Tg mouse revealed that the B220+ cells are indeed CD19+ B cells. SBM cells were isolated as noted in the Methods.

Supplementary Figure 2

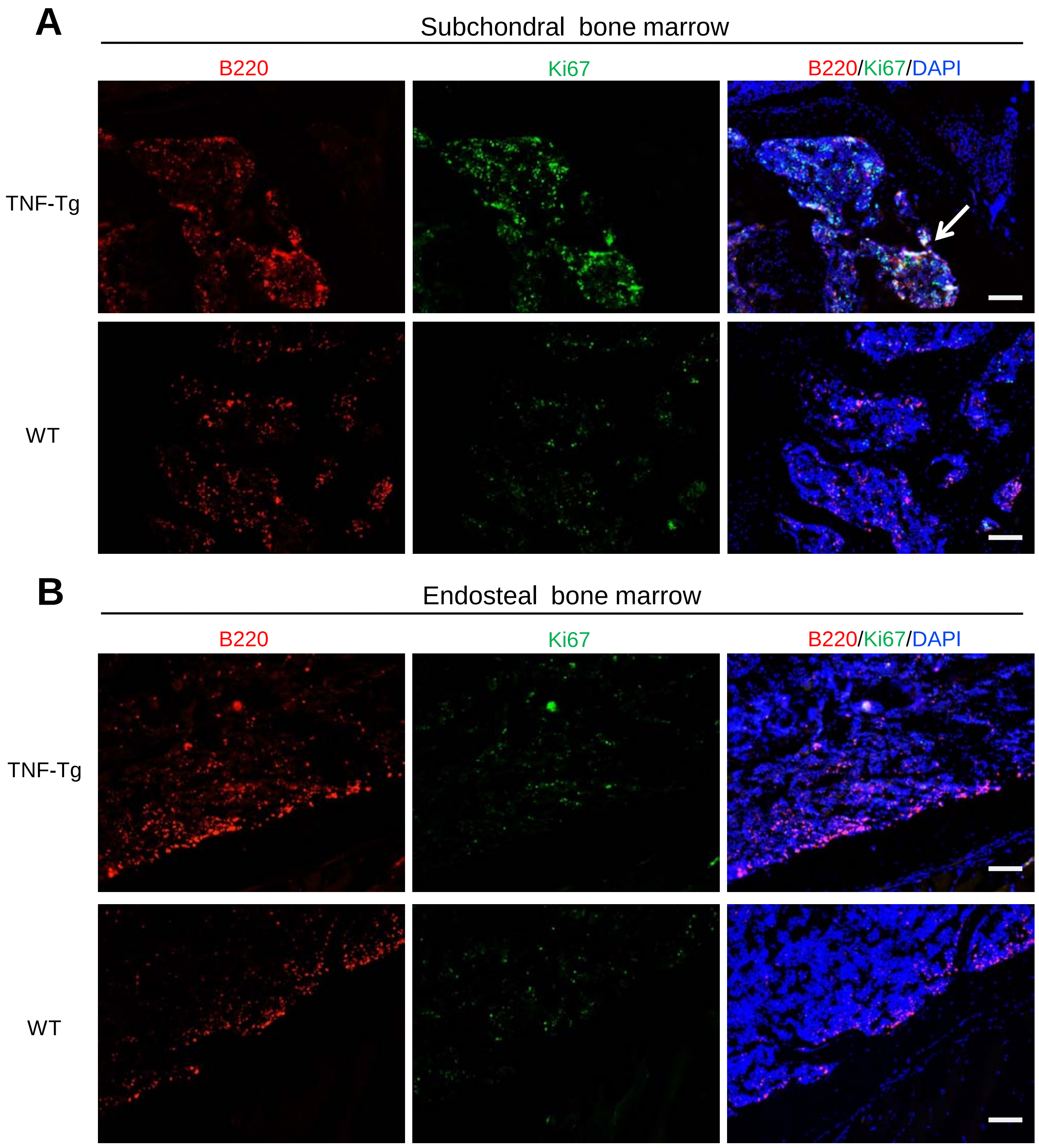

Supplementary Fig. 2. B cells in the subchondral bone marrow of TNF-Tg mice have increased proliferation.

Frozen sections of leg including knee joints from 5-m-old TNF-Tg and WT mice were subjected to IF with anti-B220 Ab for B cells (red) and anti-Ki67 Ab for cell proliferation (green). Representative images of subchondral bone marrow (**A**) and endosteal bone marrow (**B**) were shown. There are multiple areas of dual staining as highlighted by the arrow in one section. Bar =200µm. 5 mice and their controls were included in each experiment.

Supplementary Figure 3

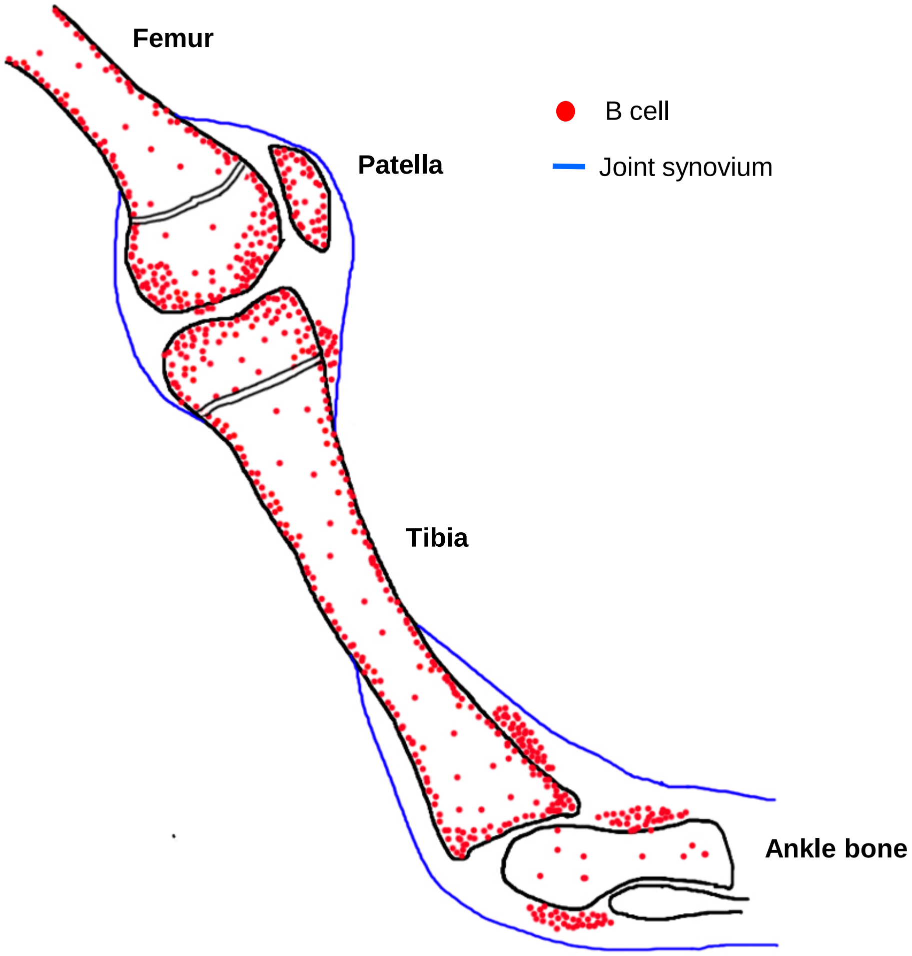

Supplementary Fig. 3. B cell distribution in long bones and joints of a TNF-Tg mouse. A cartoon showing B cell distribution in long bones and joints of a 6-m-old TNF-Tg mouse, based on IF staining with anti-B220 Ab.

Supplementary Figure 4

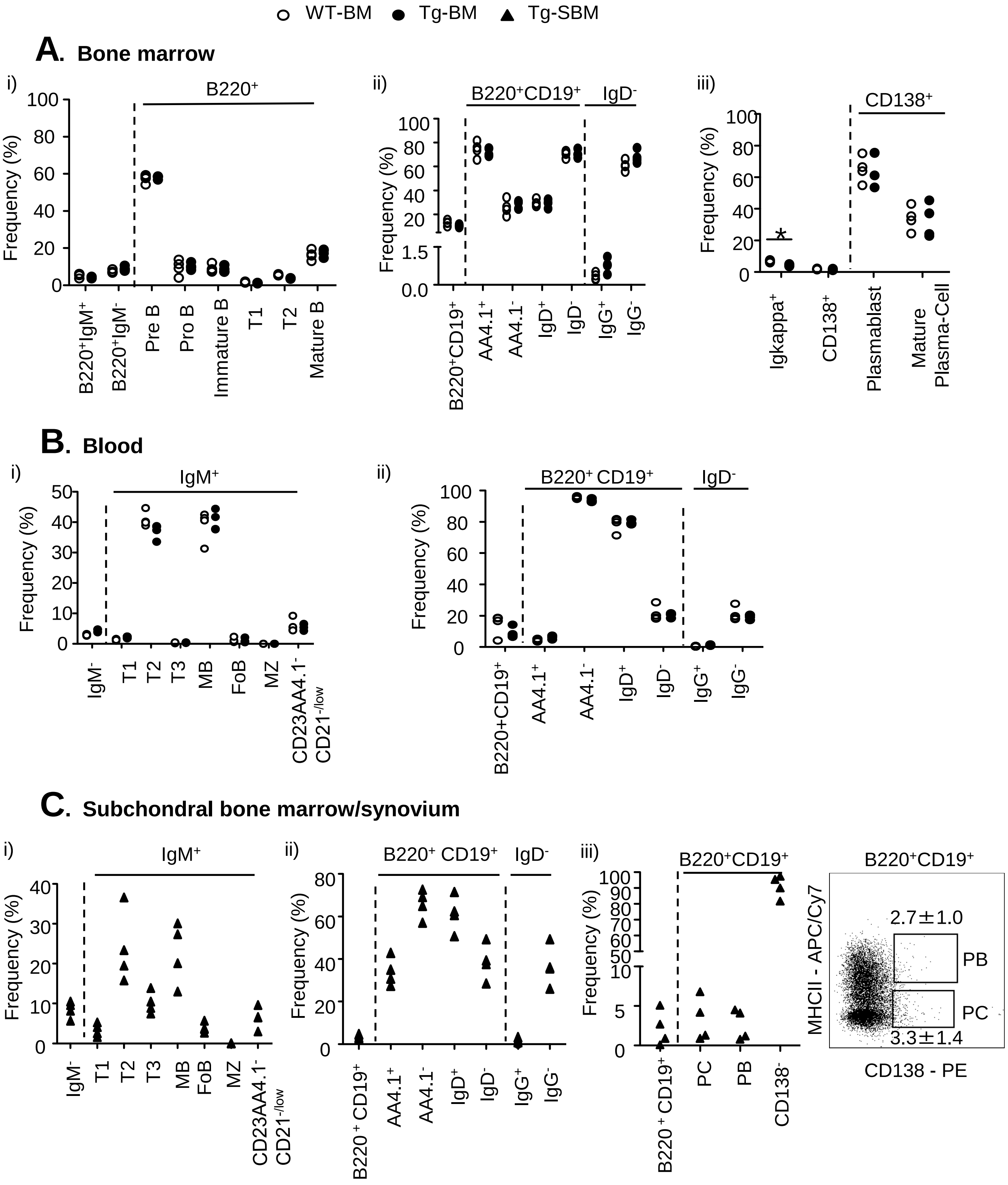

Supplementary Figure 4

D. Gating strategies used for A-i

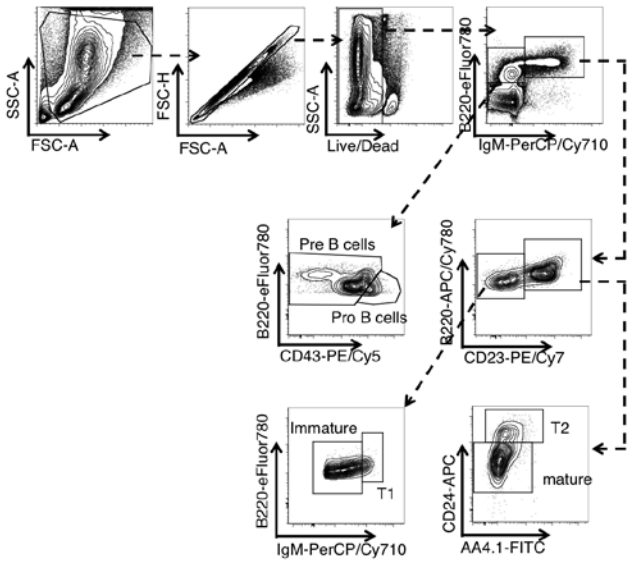

E. Gating strategies used for A-ii, B-ii, C-ii

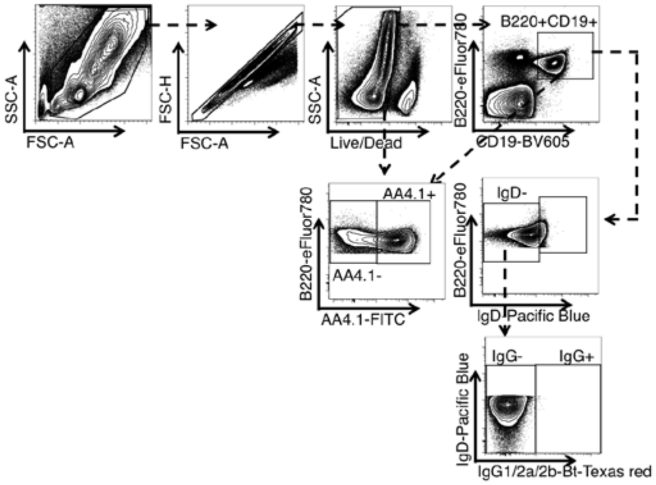

F. Gating strategies used for A-iii

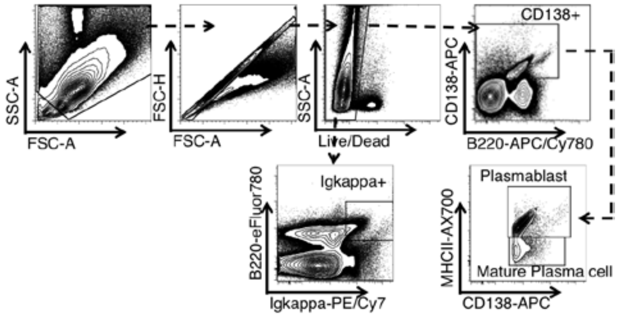

G. Gating strategies used for B-i, C-i

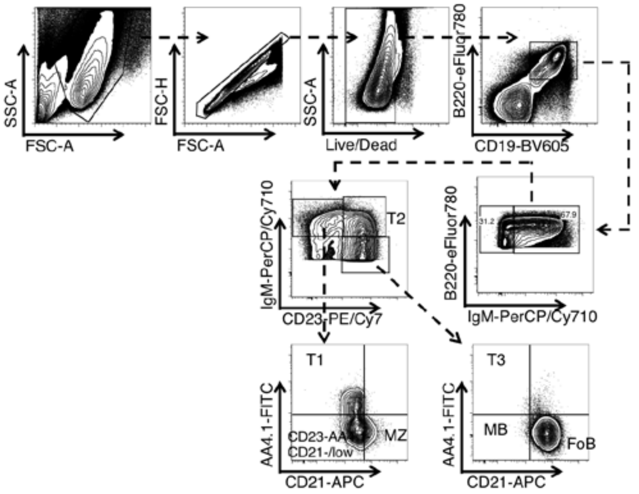

Supplementary Fig. 4. Characterization of B cells in bone marrow, blood and subchondral bone marrow/synovium of TNF-Tg mice by flow cytometry.

Total cells from bone marrow (BM), blood and subchondral bone marrow/synovium were stained with various B cell markers and the frequencies of B cell populations were enumerated by flow cytometry. 6-m-old mice were used. N=4 mice per group. (**A**) Frequency of B cell populations in the BM of TNF-Tg and WT mice. i) Frequencies of Pre B ($B220^{+}IgM^{-}CD43^{low}$), Pro B ($B220^{+}IgM^{-}CD43^{+}$), Immature B ($B220^{+}IgM^{+}CD23^{-}$), Transitional 1 ($T1=B220^{+}AA4.1^{+}IgM^{hi}CD23^{+}$), Transitional 2 ($T2=B220^{+}IgM^{+}AA4.1^{+}CD23^{+}CD24^{hi}$) and Mature B ($B220^{+}IgM^{+}AA4.1^{-}CD23^{+}CD24^{low}$) were expressed as % of $B220^{+}$ cells. ii) Frequencies of B cells based on AA4.1 or IgD expression. IgD^{-} cells were further examined for IgG expression. iii) Frequencies of $Ig\kappa^{+}$ or $CD138^{+}$ plasma cells in the BM. Plasmablast and mature plasma cell frequencies were determined within $CD138^{+}$ cells. (**B**) Frequencies of B cell populations in blood from TNF-Tg and WT mice. i) IgM^{+} B cells were analyzed for transitional 1 ($T1= B220^{+}CD19^{+}IgM^{hi}CD23^{-}AA4.1^{+}CD21^{-/low}$), transitional 2 ($T2=B220^{+}CD19^{+}IgM^{hi}CD23^{+}AA4.1^{+}CD21^{low}$), transitional 3 ($T3=B220^{+}CD19^{+}IgM^{low}CD23^{+}AA4.1^{+}CD21^{low}$), mature B cell ($MB=B220^{+}CD19^{+}IgM^{low}CD23^{+}AA4.1^{-}CD21^{low}$), follicular B cell ($FoB=B220^{+}CD19^{+}IgM^{low}CD23^{+}AA4.1^{-}CD21^{int}$) and marginal zone B cell ($MZ=B220^{+}CD19^{+}IgM^{low}CD23^{+}AA4.1^{-}CD21^{hi}$) as % of $B220^{+}CD19^{+}$ cells. ii) Frequencies of blood B cells based on AA4.1 or IgD expression. IgG expression was determined in IgD^{-} B cells. (**C**) Frequencies of B cell populations in subchondral bone marrow/synovium from TNF-Tg mice. i) Frequencies for B cell populations based on IgM expression. IgM^{+} B cells were further analyzed for B cell populations as described for blood. ii) Similar to blood, B cells from subchondral bone marrow/synovium were characterized using AA4.1 or IgD and IgG expression were examined within the IgD^{-} fraction. iii) Further characterization of SBM/synovial B cells reveals very few $CD138^{+}$ plasma cells. $B220^{+}CD19^{+}$ B cells were gated and the expression of CD138 and MHCII examined to define plasma cells ($CD138^{+}MHCII^{low}$) and plasmablasts ($CD138^{+}MHCII^{high}$). The dot plot represents the overlay of 4 separate mice, with the % +/- SEM. (**D**) Gating strategies used for A-i. (**E**) Gating strategies used for A-ii, B-ii, C-ii. (**F**) Gating strategies used for A-iii. (**G**) Gating strategies used for B-i, C-i.

Supplementary Figure 5

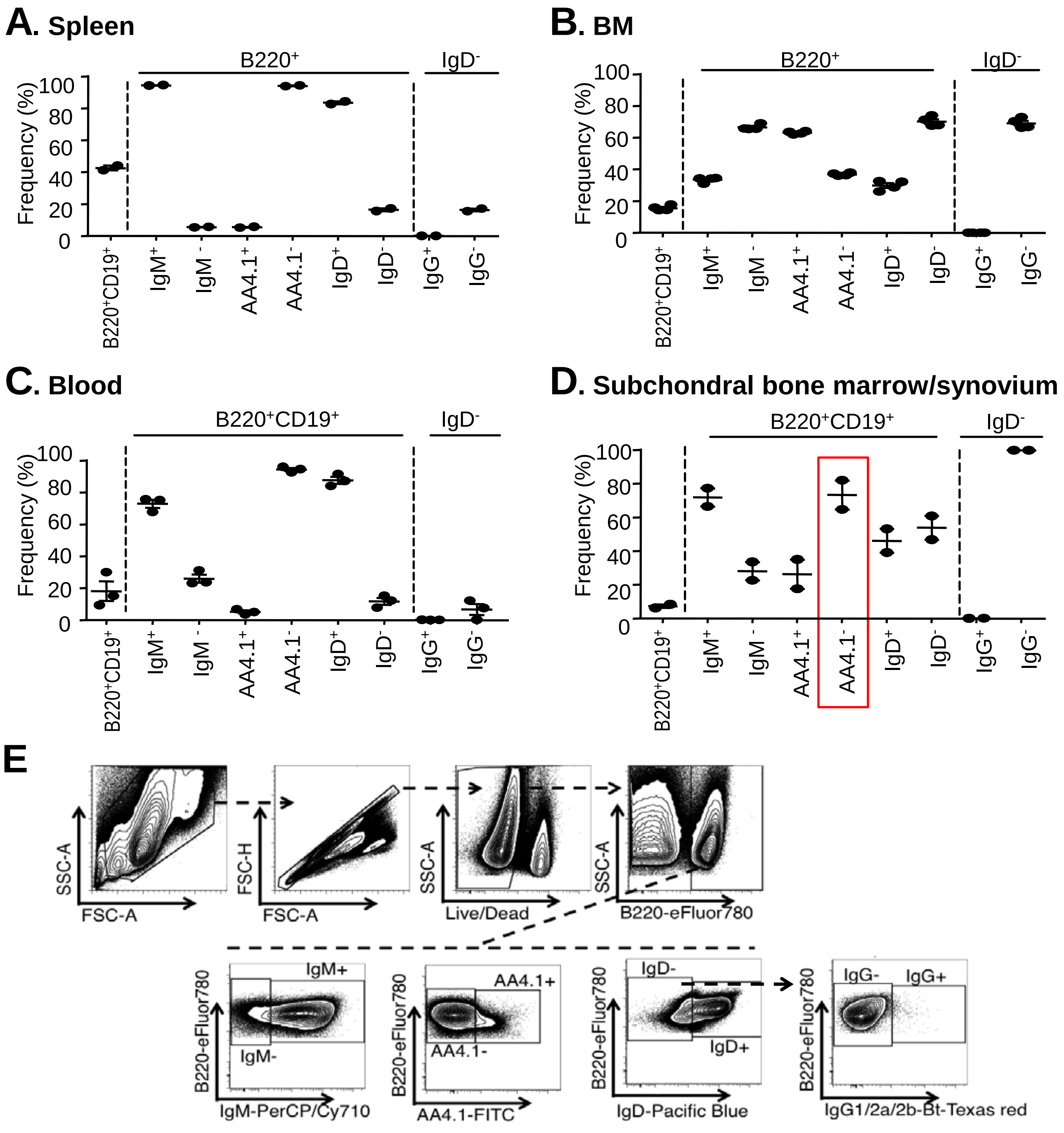

Supplementary Fig. 5. Direct comparison by flow cytometry of B cells in the TNF-Tg mouse in the spleen, blood, total bone marrow, and subchondral bone marrow /synovium.

Total cells from spleen, bone marrow (BM), blood and subchondral BM/synovium were stained with various B cell markers and the frequencies of B cell populations were enumerated by flow cytometry. 6-m-old TNF-Tg mice were used. N=2 or 3 mice per group. (**A**) Frequency of B cell populations in the spleen. (**B**) Frequency of B cell populations in the BM. (**C**) Frequency of B cell populations in the blood. (**D**) Frequency of B cell populations in the subchondral BM/synovium. The subchondral BM/synovial B cell distribution is distinct compared to total BM with an enrichment of more mature B cells (80% AA4.1$^-$ compared to 40% of the total BM B cells in the red rectangle, 50% vs. 30% IgD$^+$, 70% vs. 35% IgM$^+$). (**E**) Gating strategies used for A-D.

Supplementary Figure 6

A

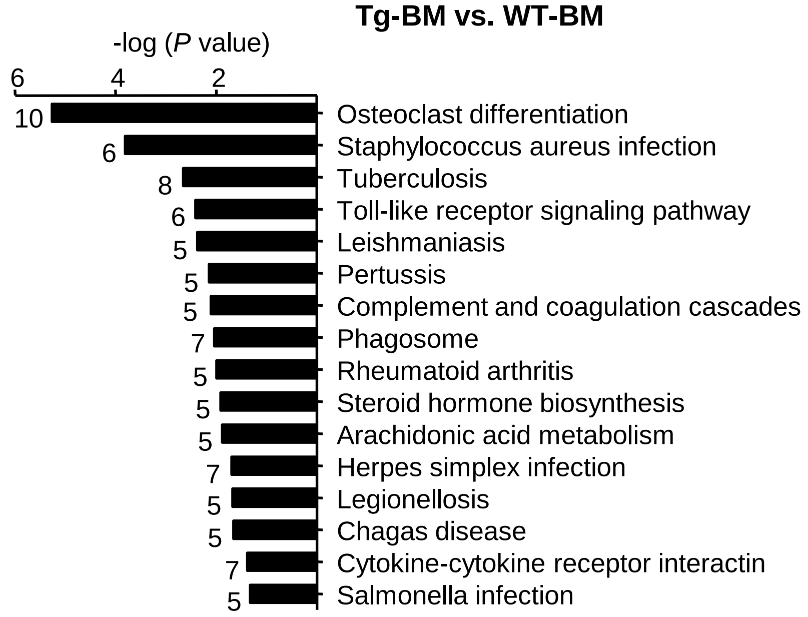

B

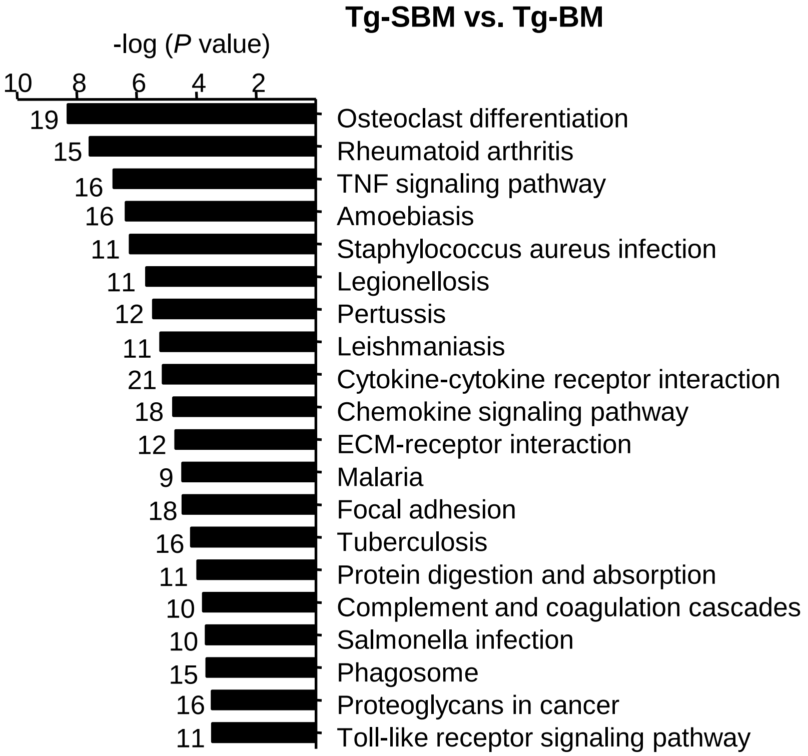

Supplementary Fig. 6. KEGG pathway analysis.

Bar graphs show the KEGG pathway analysis according to the value of − log (*p* value) for (**A**) BM comparison (Tg-BM vs. WT-BM) and (**B**) Tg comparison (Tg-SBM vs. Tg-BM); the number besides bar indicates the number of contigs concerned. BM comparison is shown for *p* value match of < 0.05 and $n \geq 5$; Tg comparison are shown for *p* value match of < 0.001 and $n \geq 5$.

Supplementary Figure 7

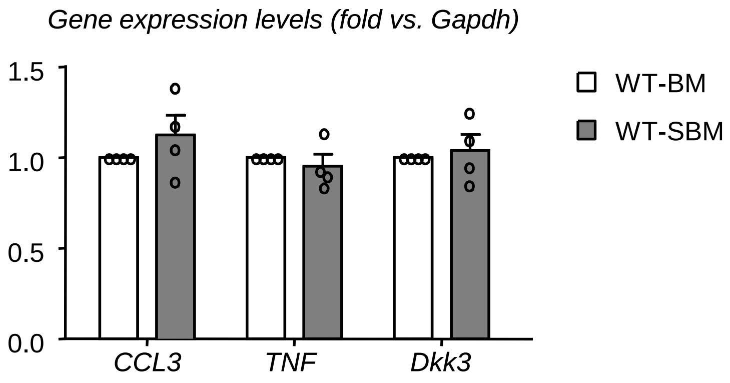

Supplementary Fig. 7. Expression of genes in B cells from WT BM vs. WT SBM.
B cells from WT BM (WT-BM) and subchondral BM (WT-SBM) were purified with magnetic beads conjugated with anti-CD19. qPCR was performed to compare the expression levels of *CCL3*, *TNF* and *Dkk3*, N=4 independent experiments.

Supplementary Figure 8

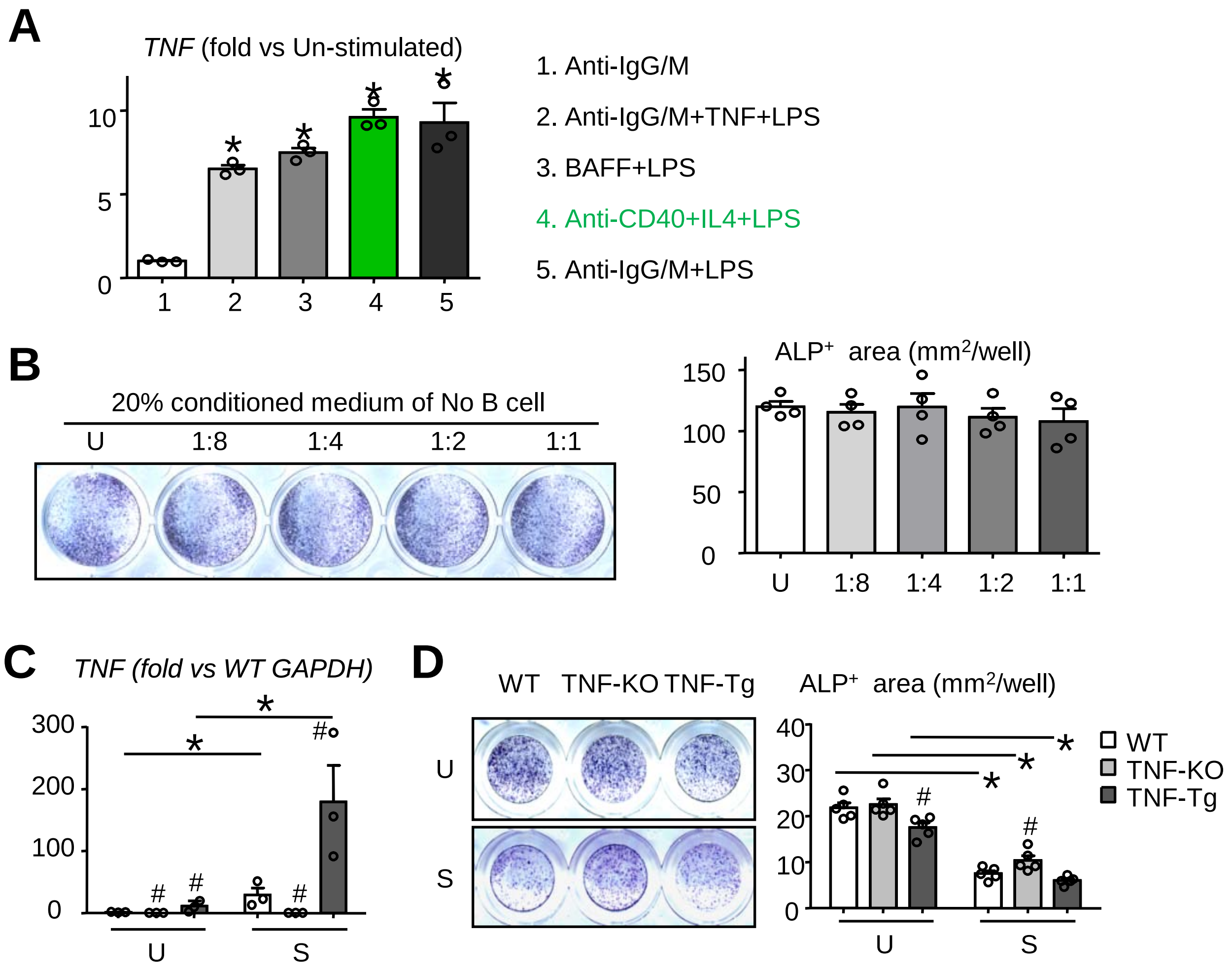

Supplementary Fig. 8. B cells inhibit osteoblast differentiation in RA partly by secreting TNF. (**A**) WT BM B cells were purified with magnetic beads conjugated with anti-CD19 Ab and cultured with various stimulatory reagents, as indicated for 48 hours. Expression of *TNF* mRNA was assessed by qPCR. Anti-CD40+IL4+LPS was the best stimulation cocktail and was used for subsequent experiments. *p<0.05 vs. 1. (**B**) B cell culture medium was added with 1/8, 1/4, 1/2 and 1/1 dilutions of optimal cocktail concentrations (2.5µg/ml Anti-CD40+10ng/ml IL4+10µg/ml LPS), but without B cells. Mesenchymal precursor cells (MPCs) were cultured in OB-inducing medium + 20% of the above B cell media for 2 days. Culture plates were stained for ALP, and ALP^{+} areas were measured. (**C**) Expression of *TNF* in stimulated (S) or un-stimulated (U) WT, TNF-KO and TNF-Tg BM B cells was measured by qPCR. Fold-changes were calculated by dividing TNF-Tg values by the value from WT cells. (**D**) Stimulated or un-stimulated WT, TNF-KO and TNF-Tg B cells were co-cultured with WT mesenchymal precursor cells in OB-inducing medium for 2 days. ALP^{+} area was measured. *p<0.05 vs. U, # p<0.05 vs. WT. Each experiment was performed 3 to 5 times. Representative image and quantifications shown come from one independent experiment. All error bars represent s.e.m. One way ANOVA followed by Dunnett's post-hoc multiple comparisons was performed.

Supplementary Figure 9

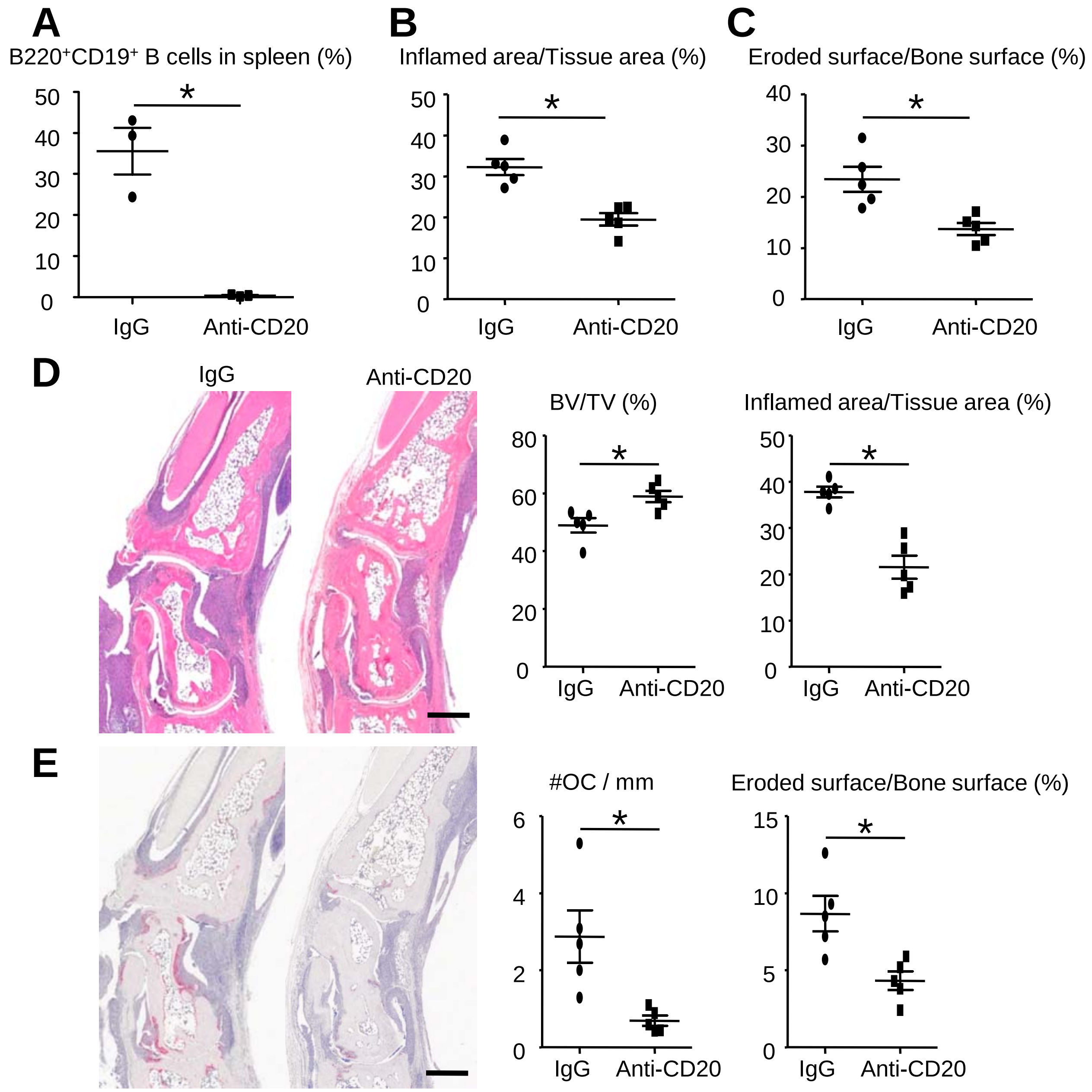

Supplementary Fig. 9. B cell depletion therapy decreases knee and ankle inflammation of TNF-Tg mice.

2.5-month-old TNF-Tg mice and WT littermates were given murine anti-CD20 Ab (10 mg/kg/injection intravenously) or IgG once a week for 8 weeks. (**A**) Frequency of B220$^+$CD19$^+$ B cells in the spleen of 3 mice was analyzed by flow cytometry. (**B**) Histomorphometric analyses of inflamed area/tissue area were performed on H&E-stained knee sections. (**C**) Eroded surface/bone surface was analyzed, based on TRAP-stained knee sections. (**D**) H&E-stained ankle sections and histomorphometric analyses of percentage of BV/TV and inflamed area/tissue area. (**E**) TRAP-stained sections, and the number of TRAP$^+$ OCs/mm bone surface and eroded surface/bone surface (%). Bar =500μm. *$p<0.05$ vs. IgG-treated mice. 5 mice and their controls were included in each experiment. These experiments were repeated once with representative images and quantifications shown. All error bars represent s.e.m. Two-tailed unpaired Student's t-test was performed.

Supplementary Figure 10

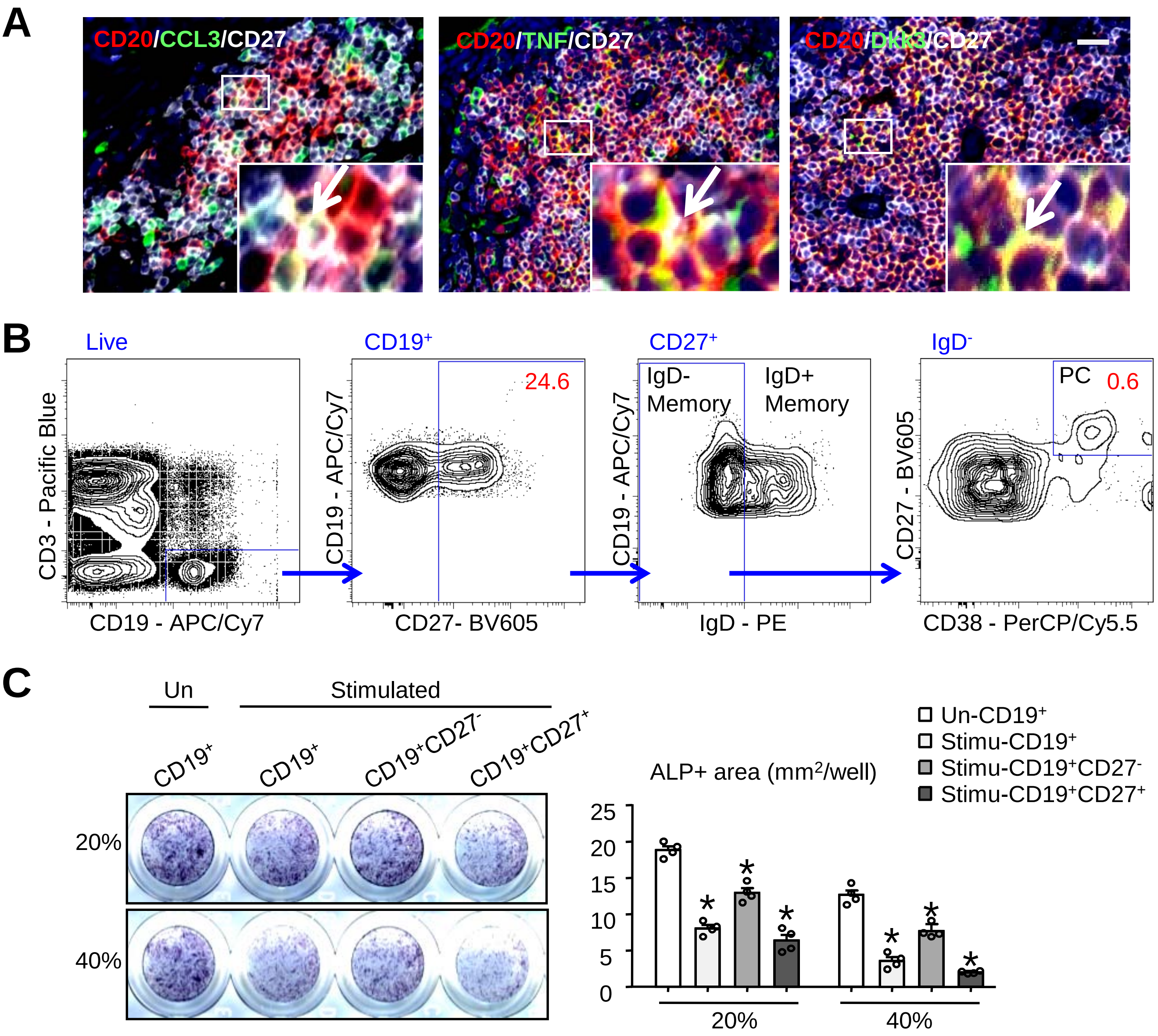

Supplementary Fig. 10. Memory B cells from RA patients express OB inhibitors and inhibit OB differentiation.

(**A**) RA synovium was stained with Abs to CD20 (B cells), CD27 (memory B cells), CCL3, TNF and DKK3. White arrows indicate the cells with triple staining. Bar =25μm. 3 patients and their controls were included. (**B**) Flow cytometry of peripheral blood B cells from an RA patient. The plots show that 24.6 % of B cells are $CD27^+$ (including $CD27^{hi}$ plasma cells). However, only a small fraction of these $CD27^+$ B cells are plasma cells, defined as $CD19^+IgD^-CD27^{hi}CD38^{hi}$ (0.6% of B cells). (**C**) Blood B cells were purified with magnetic beads conjugated with anti-CD19 Ab and anti-CD27 Ab, and cultured with CpG2006+anti-Ig(A+G+M) for 4 hours. Conditioned medium (20% or 40%) from $CD19^+$, $CD19^+CD27^-$ and $CD19^+CD27^+$ B cell cultures were added to human MSCs in OB-inducing medium for 3 days. ALP staining was performed. *$p<0.05$ vs. Un-$CD19^+$. All of these experiments were repeated at least once with representative data shown. All error bars represent s.e.m. One way ANOVA followed by Dunnett's post-hoc multiple comparisons was performed.

Supplementary Figure 11

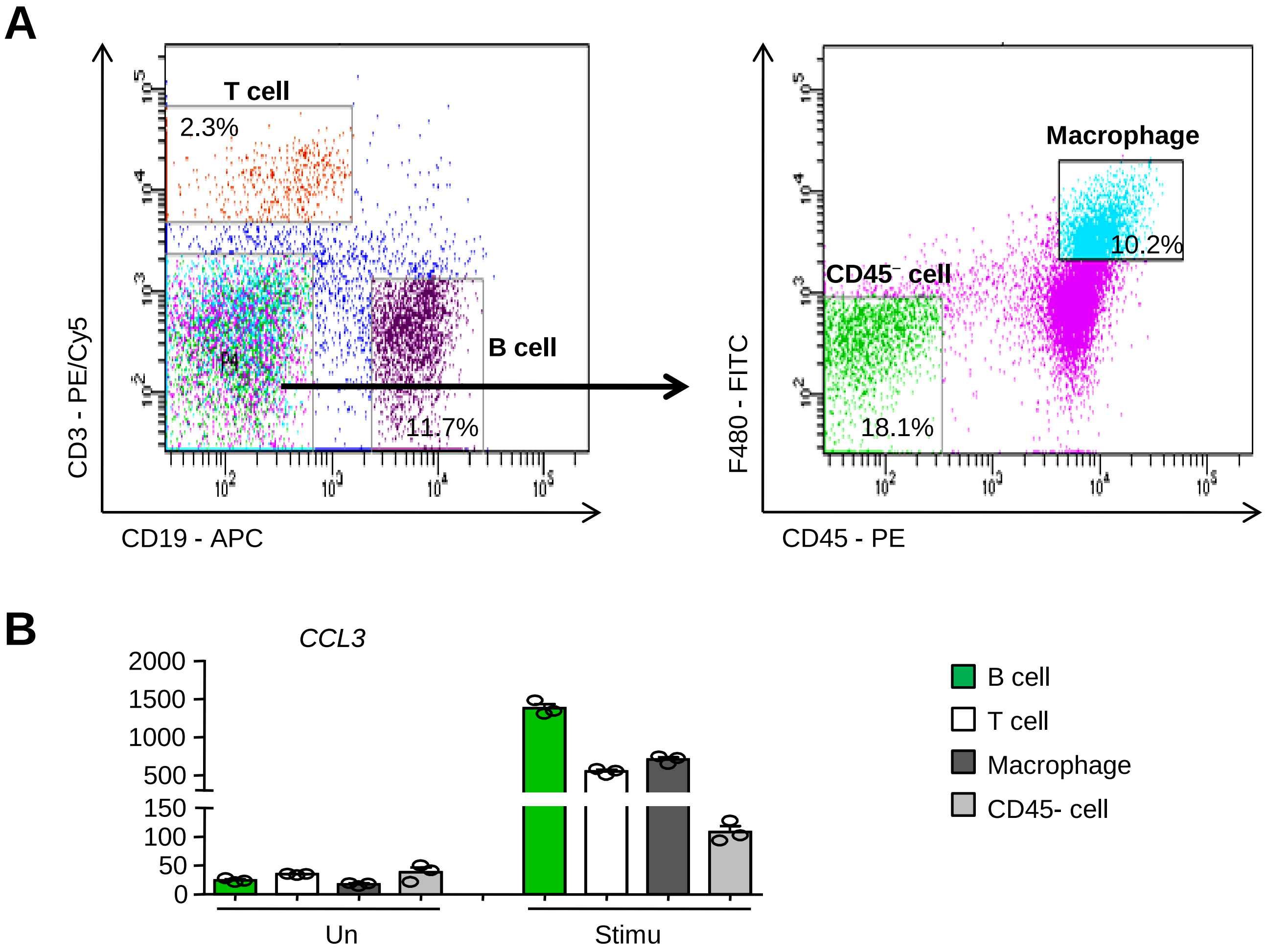

Supplementary Fig. 11. Activated B cells from normal subjects produce similar levels of CCL3 as macrophages.

Bone marrow cells from WT mice were cultured with or without the stimulatory cocktail (2.5μg/ml Anti-CD40+10ng/ml IL4+10μg/ml LPS) for 4 hours, and stained with anti-CD19 (B cell), anti-CD3 (T cell), anti-F480 (Macrophage) and anti-CD45 (for $CD45^-$ mesenchymal lineage cells) antibodies and subjected to flow cytometry sorting. (**A**) Gating strategies used for cell sorting. (**B**) Following flow cytometry sorting, RNA from all the cell types was harvested and subjected to qPCR, respectively. N=3 mice per group. Values were calculated based the equation= ½ CT (gene of interest) – CT (Gapdh) x100.

Supplementary Figure 12

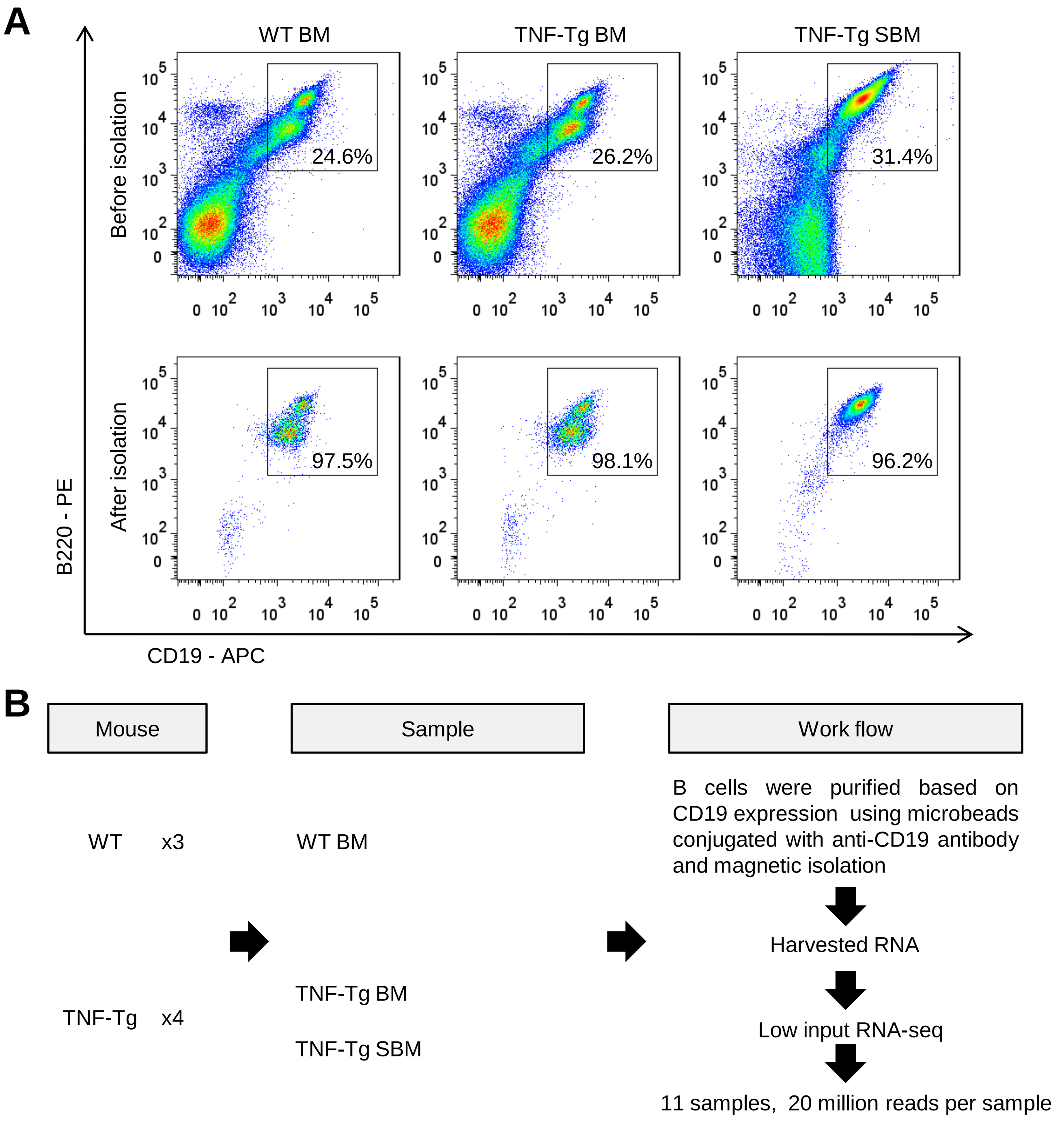

Supplementary Fig. 12. B cells were purified with anti-CD19 beads and subjected to RNA-seq. (**A**) Bone marrow (BM) cells and subchondral BM (including synovium) cells from WT or TNF-Tg mice were purified with magnetic beads conjugated with anti-CD19 Ab and analyzed by flow cytometry using anti-CD19 and anti-B220 Abs. (**B**) B cells were purified from 6-m-old WT BM (WT BM), TNF-Tg BM (TNF-Tg BM) and TNF-Tg subchondral BM including synovium (TNF-Tg SBM) with anti-CD19 beads. Following purification, RNA was harvested and subjected to low input RNA-seq. Workflow is shown. RNA-seq was performed once with 3 or 4 mice in each group.

Supplementary Figure 13

Raw data of Immunoblotting from Figure 3F.

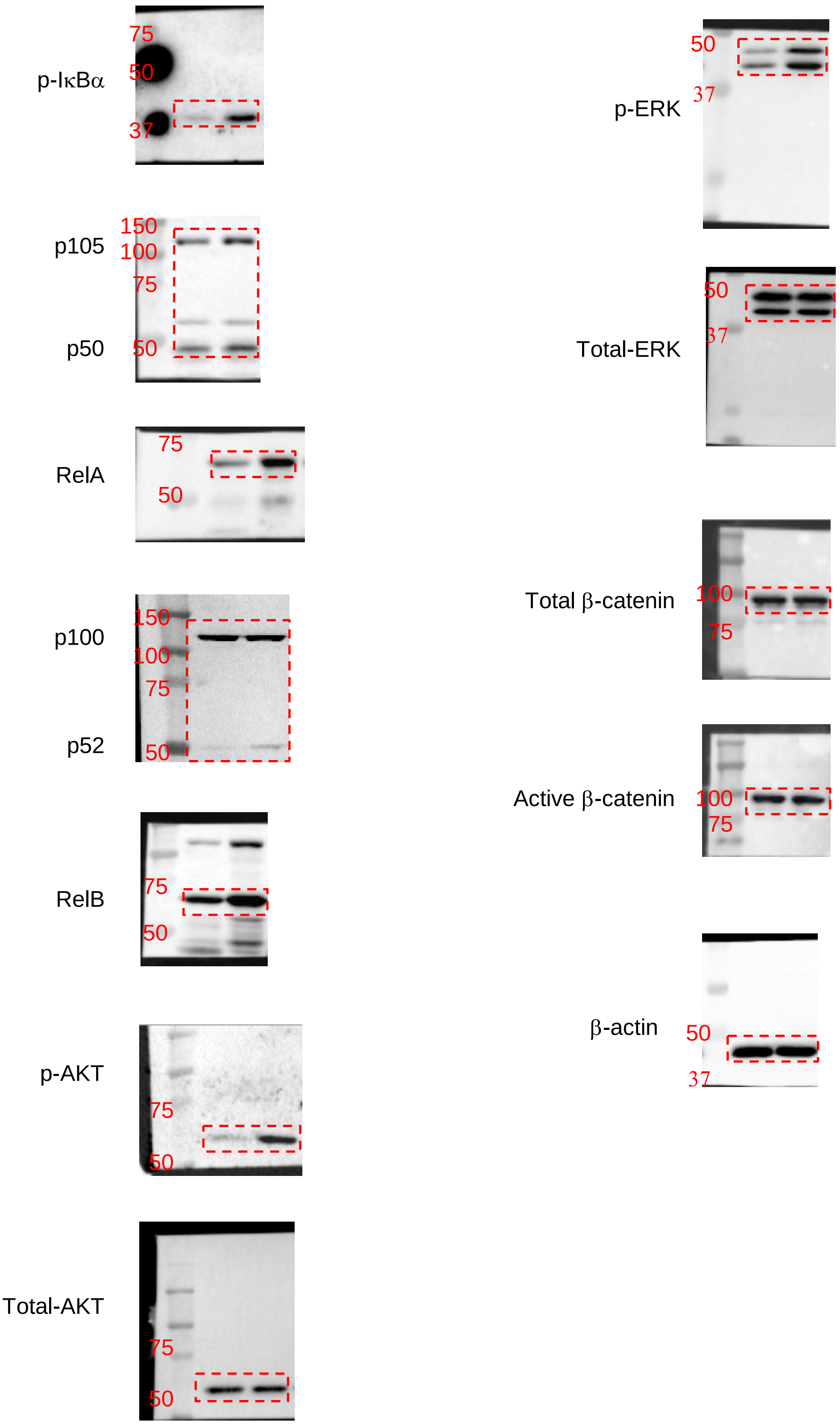

Supplementary Figure 14

Raw data of Immunoblotting from Figure 4B & C.

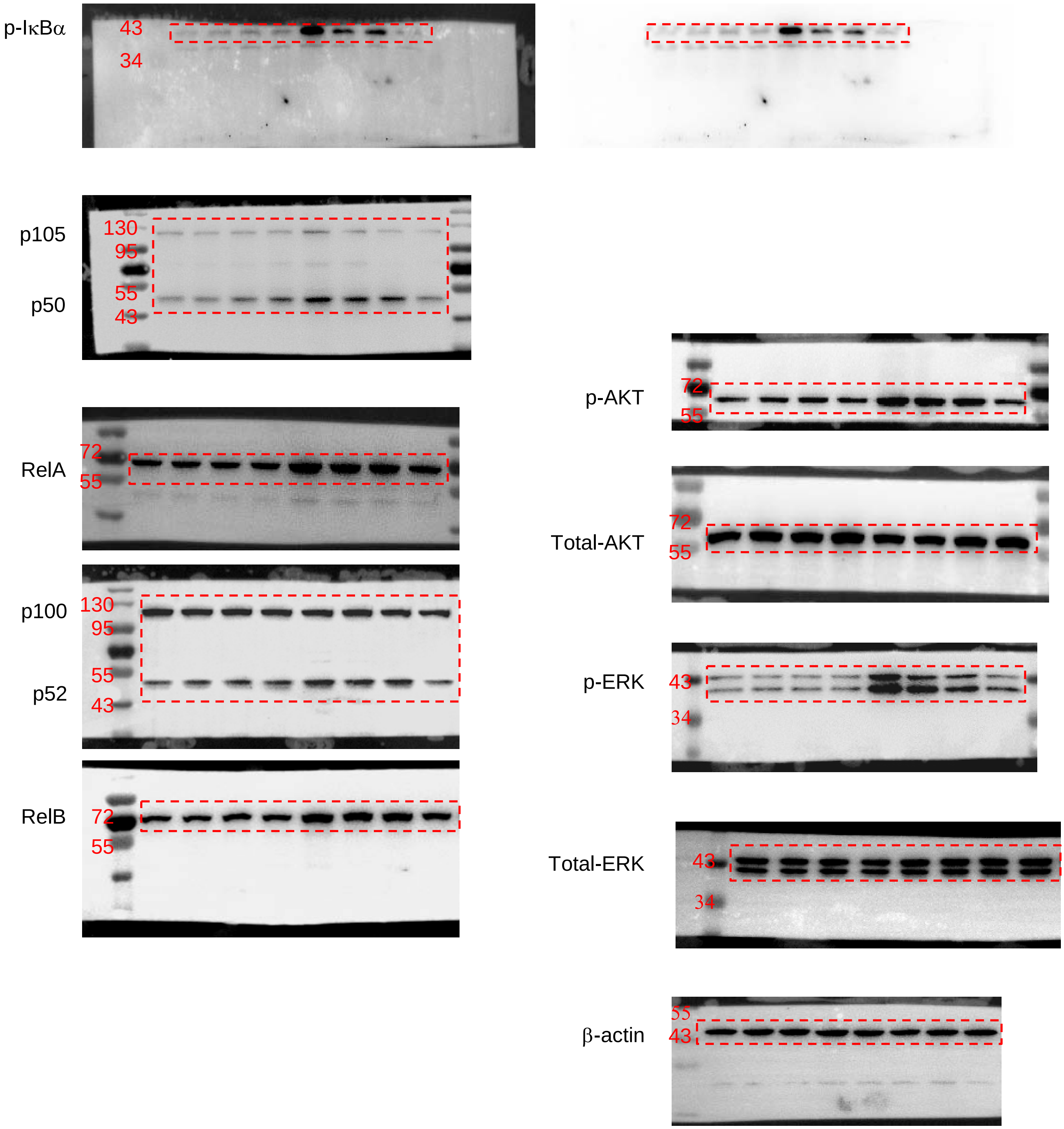

Supplementary Table 1

The 30 most differentially expressed genes for TNF-Tg SBM B cells vs. TNF-Tg BM B cells

RANK	Gene Symbol	Description	Fold Change	p value
1	Cxcl3	chemokine (C-X-C motif) ligand 3	12.25	0
2	Cxcl2	chemokine (C-X-C motif) ligand 2	11.54	0
3	Fxyd2	FXYD domain-containing ion transport regulator 2	10.50	0
4	Mmp3	matrix metallopeptidase 3	10.30	0
5	Ecm1	extracellular matrix protein 1	9.57	0
6	Cxcl1	chemokine (C-X-C motif) ligand 1	9.38	8.34E-12
7	Arg1	arginase 1	9.22	0
8	Cav1	caveolin 1	8.92	0
9	Mmp12	matrix metallopeptidase 12	8.60	3.52E-13
10	Anxa8	annexin A8	8.34	0
11	Saa3	serum amyloid A 3	8.34	0
12	Col3a1	collagen, type III, alpha 1	8.11	0
13	Dpep3	dipeptidase 3	8.09	0
14	Irg1	immunoresponsive 1 homolog	7.87	0
15	Dpep2	dipeptidase 2	7.84	0
16	Ctsk	cathepsin K	7.41	0
17	Tppp3	tubulin polymerization-promoting protein family member 3	7.21	0
**18**	**Ccl3**	**chemokine (C-C motif) ligand 3**	**7.21**	**9.84E-13**
19	Il1b	interleukin 1 beta	7.18	0
20	Ccdc109b	mitochondrial calcium uniporter dominant negative beta subunit	6.99	0
21	Cxcl13	chemokine (C-X-C motif) ligand 13	6.98	9.39E-14
22	Clec4e	C-type lectin domain family 4, member e	6.92	0
**23**	**Tnf**	**tumor necrosis factor**	**6.92**	**4.86E-14**
24	Clec4d	C-type lectin domain family 4, member d	6.77	0
25	Havcr2	hepatitis A virus cellular receptor 2	6.76	0
26	Ccl7	chemokine (C-C motif) ligand 7	6.63	8.12E-12
27	S100a4	S100 calcium binding protein A4	6.62	0
28	Spp1	secreted phosphoprotein 1	6.56	0
29	Lum	lumican	6.51	4.36E-11
30	Ccl4	chemokine (C-C motif) ligand 4	6.48	0

Supplementary Table 1. The 30 most differentially expressed genes for TNF-Tg SBM B cells vs. TNF-Tg BM B cells from RNA-seq. The genes were ranked by fold-change.

Supplementary Table 2

The 30 most differentially expressed genes for TNF-Tg BM B cells vs. WT BM B cells

RANK	Gene Symbol	Description	Fold Change	p value
1	Marco	macrophage receptor with collagenous structure	11.8115	3.52E-13
2	Liph	lipase, member H	6.4426	6.22E-06
3	Madcam1	mucosal vascular addressin cell adhesion molecule 1	5.70067	6.85E-07
4	Tppp3	tubulin polymerization-promoting protein family member 3	5.47954	6.32E-07
5	Aplnr	apelin receptor	5.20627	3.26E-06
6	Rap1gap2	RAP1 GTPase activating protein 2	4.94378	8.83E-06
7	Gpnmb	glycoprotein (transmembrane) nmb	-4.83397	0
8	Apoc1	apolipoprotein C-I	4.35723	7.38E-05
9	C3	complement component 3	4.09893	3.5E-11
10	Cd300e	CD300E molecule	3.69617	1.12E-05
11	Fpr-rs4	formyl peptide receptor, related sequence 4	3.55737	7.94E-06
12	Fpr1	formyl peptide receptor 1	3.53647	1.99E-11
13	Clec5a	C-type lectin domain family 5, member a	3.36941	4.84E-06
14	Atp6v0d2	ATPase, H+ transporting, lysosomal V0 subunit D2	-3.35399	5.87E-06
15	Ifitm6	interferon induced transmembrane protein 6	3.19212	1.65E-07
16	Hp	haptoglobin	3.15671	1.37E-10
17	Fpr3	formyl peptide receptor 3	3.04872	6.88E-07
18	Fpr2	formyl peptide receptor 2	2.98473	3.55E-07
19	Mrgpra6	MAS-related GPR, member A6	2.96882	4.04E-05
20	Mrgpra9	Mrgpra9 MAS-related GPR, member A9	2.96618	1.66E-05
21	Lcn2	lipocalin 2	2.9627	1.58E-08
22	Mrgpra2a	Mrgpra2a MAS-related GPR, member A2A	2.94803	2.62E-06
23	Mrgpra1	Mrgpra1 MAS-related GPR, member A1	2.92765	1.69E-05
24	Mrgpra2b	Mrgpra2b MAS-related GPR, member A2B	2.92626	2.94E-06
25	Mrgpra3	MAS-related GPR, member A3	2.88094	5.92E-05
26	Mrgpra4	Mrgpra4 MAS-related GPR, member A4	2.87229	3.35E-05
27	Chi3l3	chitinase-like 3	2.75127	1.04E-07
28	Vsig4	V-set and immunoglobulin domain containing 4	2.6962	3.53E-06
29	Chi3l4	chitinase 3-like 4	2.6958	2.03E-07
30	Cd177	CD177 antigen	2.64779	1.76E-07

Supplementary Table 2. The 30 most differentially expressed genes for TNF-Tg BM B cells vs. WT BM B cells from RNA-seq. The genes were ranked by fold-change.

Supplementary Table 3

Sequence of qPCR primers.

Name	F/R	Sequences
m*CCL3*	F	5’ AAGGATACAAGCAGCAGCGAGTA 3’
	R	5’ TGCAGAGTGTCATGGTACAGAGAA 3’
m*TNF*	F	5’ CACACTCAGATCATCTTCTCAA 3’
	R	5’ AGTAGACAAGGTACAACCCATC 3’
m*Dkk3*	F	5’ CTCGGGGGTATTTTGCTGTGT 3’
	R	5’ TCCTCCTGAGGGTAGTTGAGA 3’
m*Runx2*	F	5’CAAGAAGGCTCTGGCGTTTA3’
	R	5’TGCAGCCTTAAATGACTCGG3’
m*ALP*	F	5’CTTGCTGGTGGAAGGAGGCAGG3’
	R	5’CACGTCTTCTCCACCGTGGGTC3’
m*Gapdh*	F	5’ GGTCGGTGTGAACGGATTTG 3’
	R	5’ ATGAGCCCTTCCACAATG 3’
h*CCL3*	F	5’ AGTTCTCTGCATCACTTGCTG 3’
	R	5’ CGGCTTCGCTTGGTTAGGAA 3’
h*TNF*	F	5’ CCTCTCTCTAATCAGCCCTCTG 3’
	R	5’ GAGGACCTGGGAGTAGATGAG 3’
h*Runx2*	F	5’ TCAACGATCTGAGATTTGTGGG 3’
	R	5’ GGTCAAGGTGAAACTCTTGCC 3’
h*ALP*	F	5’ AACATCAGGGACATTGACGTG 3’
	R	5’ GTATCTCGGTTTGAAGCTCTTCC 3’
h*Gapdh*	F	5’ AAGGTGAAGGTCGGAGTCAAC 3’
	R	5’ GGGGTCATTGATGGCAACAATA 3’
